# Treatment Associated Changes of Functional Connectivity of Midbrain/Brainstem Nuclei in Major Depressive Disorder

**DOI:** 10.1038/s41598-017-09077-5

**Published:** 2017-08-17

**Authors:** Gerd Wagner, Feliberto de la Cruz, Stefanie Köhler, Karl-Jürgen Bär

**Affiliations:** Psychiatric Brain and Body Research Group Jena, Department of Psychiatry and Psychotherapy, University Hospital Jena, Philosophenweg 3, Jena, 07743 Germany

## Abstract

Previous functional magnetic resonance imaging (fMRI) studies demonstrated an abnormally coordinated network functioning in Major Depression Disorder (MDD) during rest. The main monoamine-producing nuclei within midbrain/brainstem are functionally integrated within these specific networks. Therefore, we aimed to investigate the resting-state functional connectivity (RSFC) of these nuclei in 45 MDD patients and differences between patients receiving two different classes of antidepressant drugs. Patients showed reduced RSFC from the ventral tegmental area (VTA) to dorsal anterior cingulate cortex (dACC) and stronger RSFC to the left amygdala and dorsolateral prefrontal cortex (DLPFC). Patients treated with antidepressants influencing noradrenergic and serotonergic neurotransmission showed different RSFC from locus coeruleus to DLPFC compared to patients treated with antidepressants influencing serotonergic neurotransmission only. In the opposite contrast patients showed stronger RSFC from dorsal raphe to posterior brain regions. Enhanced VTA-RSFC to amygdala as a central region of the salience network may indicate an over‐attribution of the affective salience to internally-oriented processes. Significant correlation between decreased VTA-dACC functional connectivity and the BDI-II somatic symptoms indicates an association with diminished volition and behavioral activation in MDD. The observed differences in the FC of the midbrain/brainstem nuclei between two classes of antidepressants suggest differential neural effects of SSRIs and SNRIs.

## Introduction

The psychopathology of major depressive disorder (MDD) is characterized by an increased negative affect, feelings of worthlessness, anhedonia and increased negative self-referential processing. The brain network, which processes self-related information, is remarkably similar to another network: the default mode network (DMN) with the core regions: posterior cingulate cortex (PCC) and the ventromedial prefrontal cortex (VMPFC)^1^. Activity was described in the DMN when thoughts are directed towards internal processes (“mind wandering”). In contrast, brain regions of the DMN decrease activity (“deactivated”) during processing of external stimuli, e.g. cognitive tasks. Functional activation was then observed in brain regions of the “task-positive” executive control network (ECN)^2^. The ECN encompasses the lateral prefrontal, e.g. dorsolateral prefrontal cortex (DLPFC), parietal and cerebellar regions and typically shows a strong negative correlation (“anticorrelation”) to regions of the DMN^1^. Using resting-state fMRI (rs-fMRI), Hamilton, *et al*.^3^ provided evidence for an abnormal interaction between the DMN and the ECN network in MDD. A recent meta-analysis of rs-fMRI studies in MDD revealed in agreement with this notion a hypoconnectivity within the ECN and a hyperconnectivity within the DMN network as well as abnormal functional connectivity (FC) between regions of these networks^4^. Moreover, this meta-analysis indicated an abnormally coordinated network functioning between DMN, ECN and a third network, the so-called “salience network”. The salience network is anchored by dorsal anterior cingulate (dACC) and the insular cortices, but also by the ventral tegmental area (VTA), substantia nigra, the amygdala and ventral striatum^2^. It has been shown to activate in response to different forms of motivational salience^2^. Pharmacological studies demonstrate that activity within the DMN is influenced by dopaminergic (DA), noradrenergic (NA), and serotonergic (5-HT) neurotransmission^5–9^. In our recent study, we used rs-fMRI and graph theoretical analysis to elucidate the resting-state functional connectivity (RSFC) and network organization of the monoamine-producing midbrain/brainstem nuclei in a large sample of healthy subjects^10^. We showed that serotonergic brainstem nuclei, i.e. nucleus raphes dorsalis (DRN) and nucleus centralis superior (NCS) as well as the dopaminergic ventral tegmental area (VTA) and substantia nigra pars compacta (SNc) are functionally integrated within the DMN. Additionally, an independent component analysis (ICA) also revealed the participation of the DA nuclei in the salience network, indicating their wide-ranging connectivity. In contrast to 5-HT and DA nuclei, the noradrenergic locus coeruleus (LC) was part of the ECN.

The clinically relevant monoamine-deficiency theory postulates that the pathophysiology of MDD is associated with a deficiency of the monoamine neurotransmitters serotonin, norepinephrine and/or dopamine in the central nervous system. In addition, it is assumed that antidepressants exert their therapeutic action by increasing extracellular availability of monoamines, particularly at synaptic level^11^. This hypothesis emerged largely from the observations that reserpine depletes vesicular monoamine stores and reduces mood as well as from the effect of monoamine oxidase inhibitors (MAOIs)^12^.

There is strong evidence to assume that the neural circuitry for emotion regulation and social cognition, which strongly relies on the amygdala and distinct medial prefrontal regions, is serotonergically modulated^13, 14^, whereas the reward processing neural network, anchored by the VTA, the ventral striatum and the medial prefrontal cortex, is dopaminergically modulated^15^. Alterations in these neural circuits might be associated with different symptoms in MDD such as persistent low mood or anxiety (5-HT associated) or psychomotor speed, apathy and anhedonia (DA associated). Furthermore, the LC-NA system is considered to play a central role for attention shifting and cognitive flexibility^16^ as well as for central stress responses^17^. Thus, often observed cognitive deficits in depressed patients, in particular regarding executive functions, might be related to alteration in the LC-NA system.

Therefore, we hypothesized in the present study altered functional connectivity (FC) of the 5-HT and DA nuclei with DMN and salience network regions in MDD. We also hypothesized different FC of the midbrain/brainstem nuclei depending on the antidepressant treatment. Thus, we aimed to investigate, whether patients receiving antidepressant drugs modulating the 5-HT neurotransmission (SSRI) differ with regard to the RSFC from the midbrain/brainstem nuclei to patients receiving drugs influencing the 5-HT as well as NA neurotransmission (SNRI/NaSSA).

## Results

The functional connectivity analyses were carried out by correlating the regional time course, which was extracted from the selected midbrain/brainstem ROIs, against all other voxels within the brain. The functional connectivity was obtained by computing Pearson correlation coefficients. To improve the normalization of the brainstem/midbrain and to more precisely define regions of interest for the subsequent time-series extraction the spatially unbiased infra-tentorial template (SUIT, version 3.1)^18^ was used. The brainstem/midbrain ROIs were defined according to known localization in the anatomical literature^19^ and by comparison with available atlases of the human brainstem^20^. The time series were extracted from the unsmoothed with SUIT normalized functional brainstem/cerebellum images.

### Resting state functional connectivity in the total MDD group

#### 5-HT nuclei

Comparing differences in the FC of the DRN and NCS, we only detected a significantly stronger RSFC in patients with MDD compared to controls from the NCS to the left (x = −60, y = −30, z = 0, t = 6.9, p < 0.001 uncorr., cluster extent = 249, p < 0.05 FDR corrected) and right middle temporal gyrus (x = 64, y = −12, z = −8, t = 4.3, p < 0.001 uncorr., k_e_ = 50, p < 0.05 FDR corrected; x = 52, y = −32, z = −10, t = 4.4, p < 0.001 uncorr., k_e_ = 42, p < 0.05 FDR corrected). Furthermore, a significantly stronger negative (“anticorrelated”) FC was detected in healthy controls compared to MDD patients from NCS to a cluster lying in the paracentral lobule (x = −2, y = −24, z = 64, t = 4.1, p < 0.001 uncorr., k_e_ = 50, p < 0.05 FDR corrected).

#### Noradrenergic LC

Testing for differences in the LC-RSFC, a significantly stronger connectivity was observed in controls from the LC to the left cerebellum (x = −36, y = −70, z = −50, t = 4.7, p < 0.001 uncorr., k_e_ = 74, p < 0.05 FDR corrected). In the opposite contrast, MDD revealed stronger FC than healthy controls from LC to the right superior temporal gyrus (x = 60, y = 2, z = −2, t = 4.3, p < 0.001 uncorr., k_e_ = 144, p < 0.05 FDR corrected) as well as to three clusters located on the postcentral gyrus (x = −52, y = −18, z = 56, t = 4.4, p < 0.001 uncorr., k_e_ = 43, p < 0.05 FDR corrected), on the precentral gyrus (x = −30, y = −26, z = 68, t = 3.9, p < 0.001 uncorr., k_e_ = 41, p < 0.05 FDR corrected) and in the SMA (x = −4, y = −24, z = 56, t = 4.1, p < 0.001 uncorr., k_e_ = 40, p < 0.05 FDR corrected). Considering the direction of this difference, MDD patients exhibited stronger positive RSFC, whereas healthy controls showed negative (“anticorrelated”) functional connectivity from LC to these regions.

#### Dopaminergic midbrain nuclei

**Figure 1:**
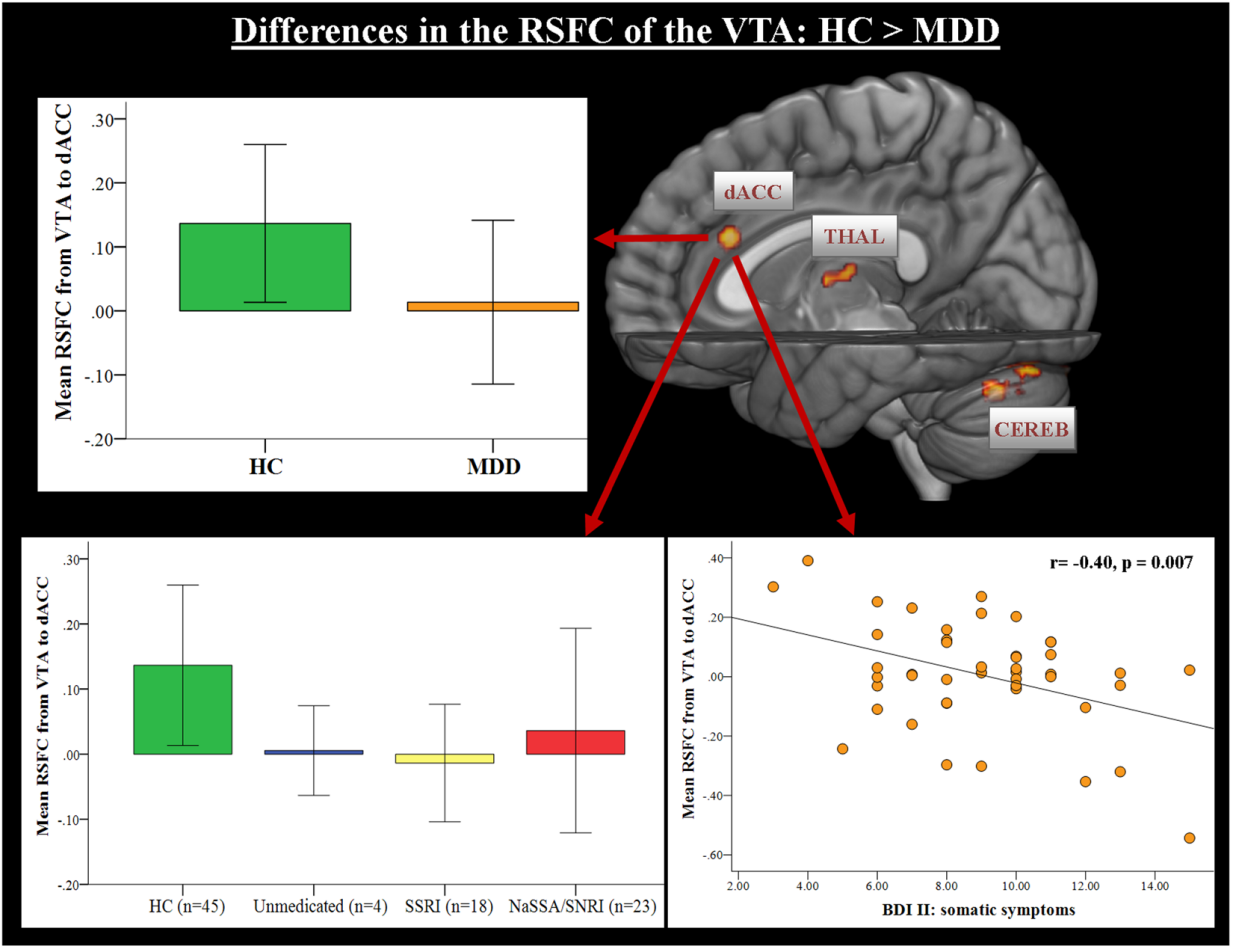
Whole-brain resting-state functional connectivity maps with seed region in the ventral tegmental area (VTA). In the upper part of the figure, the comparison between healthy controls vs. depressed patients demonstrated that patients had weaker functional connectivity from VTA to dACC, mediodorsal thalamus and bilateral cerebellum. The average BOLD time course of the voxels within the VTA was extracted for the FC analysis from the unsmoothed midbrain/brainstem functional data, which were normalized using the SUIT toolbox and DARTEL approach. In the lower left part of the figure, functional connectivity from VTA to dACC is shown split according to the class antidepressant treatment. The error bars in the graphs of the RSFC from VTA to dACC represent standard deviation. In the lower right part of the figure, a significant correlation between the functional connectivity from the VTA to dACC and BDI-II somatic symptoms in patients with MDD is depicted. The somatic factor as computed according to Vanheule, *et al*.^22^ is composed of BDI-II items such as fatigue, appetite disturbance, loss of sexual interest and concentration difficulties. Abbr.: dACC, dorsal anterior cingulate cortex; PCC, posterior cingulate cortex; THAL, thalamus; CEREB, cerebellum; SSRI, selective serotonin reuptake inhibitors; SNRI, serotonin and noradrenalin reuptake inhibitors; NaSSA, noradrenergic and specific serotonergic antidepressants.

As illustrated in Fig. 1 and Table 1, a significantly reduced connectivity in MDD compared to healthy controls was observed from VTA to dACC, to the mediodorsal thalamus and to four clusters lying in the left and right cerebellum. Due to the crucial role of dACC in MDD^21^, we correlated the abnormal RSFC between the VTA and dACC with the BDI-II total score as well as with three BDI-II subscales assessing a somatic, affective, and cognitive dimension. The computation of the subscales was based on the factor-structure model of Vanheule, *et al*.^22^. As illustrated in Fig. 1, a significantly negative correlation was detected between the BDI-II somatic subscale and the VTA-dACC functional connectivity (r = −0.40, p = 0.007). Similarly, reduced FC was observed in MDD patients from SNc to dACC and to the left cerebellum (Table 1). The opposite contrast (MDD vs. HC) revealed increased RSFC from VTA to the cluster located in the left amygdala/hippocampus and to the left DLPFC in MDD compared to healthy controls (Fig. 1, Table 1). Again due to importance of these brain structures in the pathophysiology of MDD^23^, we correlated the VTA-amygdala and VTA-DLPFC functional connectivity with the BDI-II total as well as with the BDI-II subscale scores, but we did not detect any significant correlations.

**Table 1:** Comparison of the resting-state functional connectivity between patients with MDD and healthy controls with seed regions in the dopaminergic ventral tegmental area (VTA) as well as substantia nigra pars compacta (SNc).

Region of activation	Right/Left	Brodmann’s Area	Cluster size	MNI coordinate			T value
RSFC of the VTA: HC > MDD, p < 0.001 uncorr., cluster level: p < 0.05 FDR corr.							
				**x**	**y**	**z**	
Dorsal ACC	R	32	55	2	28	24	5.0
Mediodorsal thalamus	R/L		67	−2	−16	10	4.6
Cerebellum	L		150	−50	−64	−30	5.4
Cerebellum	R		73	52	−64	−26	5.1
Cerebellum	L		61	−12	−84	−24	4.3
Cerebellum	L		43	−30	−76	−42	4.7
**RSFC of the VTA: MDD > HC, p < 0.001 uncorr., cluster level: p < 0.05 FDR corr**.							
Precentral gyrus	L	4	1493	−36	−30	62	6.1
Postcentral gyrus	L	2		−44	−36	62	5.8
Precentral gyrus	R	4	1676	46	−16	58	5.9
Postcentral gyrus	R	2		42	−24	62	5.5
Precentral gyrus	R	4	27	24	−34	78	4.5
Precentral Gyrus	L	6	26	−60	2	16	4.0
Paracentral lobule	L	6	589	−6	−30	62	5.0
Inferior frontal gyrus	L	45	54	−54	30	4	5.0
Inferior frontal gyrus	L	46	60	−58	24	14	5.0
Inferior frontal gyrus	R	45	42	58	24	10	4.5
Amygdala/Hippocampus	L		46	−30	−12	−18	5.0
Parahippocampal gyrus	L	30	24	−20	−54	6	4.2
Insula	L	13	109	−40	−10	8	5.0
Insula	L	13	22	−40	−38	18	3.9
Insula	R	13	28	38	−4	10	3.7
Superior Temporal Gyrus	L	42	32	−62	−10	14	3.9
Superior Temporal Gyrus	L	38	22	−34	10	−18	3.9
Middle Temporal Gyrus	L	22	22	−40	−60	12	3.7
Fusiform gyrus	R	37	32	38	−56	−16	4.6
Pons	R		30	0	−24	−32	3.7
Occipital lobe	R	19	89	16	−56	−2	3.9
Occipital lobe	R	19	231	14	−80	32	4.0
Occipital lobe	L	19	206	−14	−84	30	4.6
Occipital lobe	R	19	28	30	−68	−8	4.0
**RSFC of the SNc: HC > MDD, p < 0.001 uncorr., cluster level: p < 0.05 FDR corr**.							
Dorsal ACC	R	32	81	2	28	20	4.8
Cerebellum	L		123	−28	−88	−30	4.1
Cerebellum	L		38	−42	−76	−24	4.6
**RSFC of the SNc: MDD > HC, p < 0.001 uncorr., cluster level: p < 0.05 FDR corr**.							
Postcentral gyrus	R	3	1601	40	−24	52	5.9
Precentral gyrus	R	4		32	−22	48	5.1
Postcentral gyrus	L	3	1572	−48	−22	52	5.3
Precentral gyrus	L	4		−60	−16	36	5.1
Precentral gyrus	L	4	113	−58	−4	16	5.0
Paracentral lobule	R	6	903	6	−26	68	5.1
Insula	R	13	69	36	−10	14	3.8
Insula	L	13	49	−44	−20	12	5.3
Inferior frontal gyrus	L	45	31	−58	20	16	3.7
Superior Temporal Gyrus	R	42	34	66	−8	12	4.5
Superior Temporal Gyrus	R	22	46	64	2	−2	4.4
Parahippocampal gyrus	L	37	42	−26	−52	−6	4.2
Occipital lobe	L	19	59	−28	−86	8	4.8
Occipital lobe	L	19	262	−12	−92	22	4.8
Occipital lobe	L	19/37	222	−42	−70	−2	4.0

**Figure 2:**
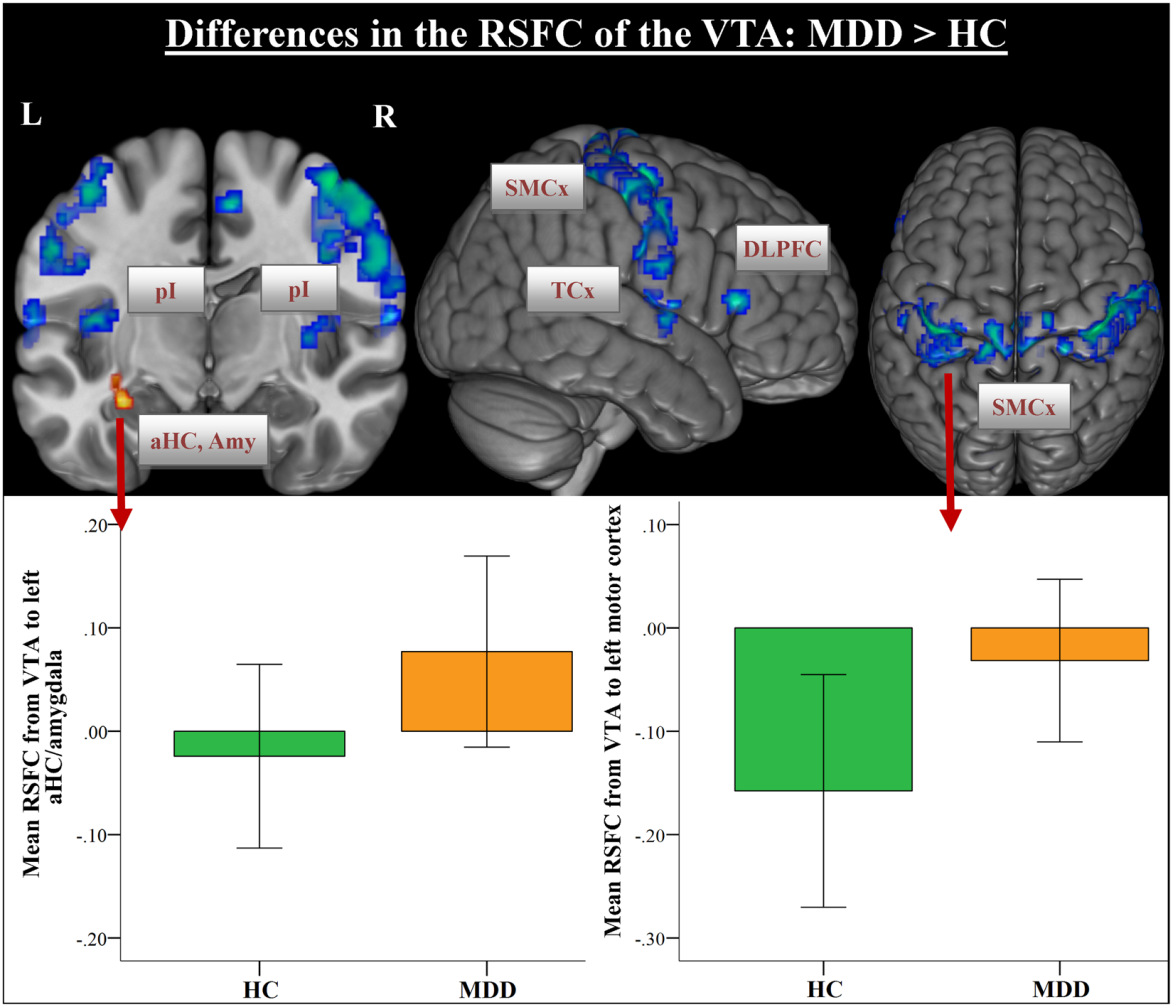
Whole-brain resting-state functional connectivity maps with seed region in the ventral tegmental area (VTA). The comparison between depressed patients vs. healthy controls demonstrated that patients had stronger functional connectivity from VTA to the left amygdala (yellow color) and absent “anti-correlation” from VTA to the sensorimotor and temporal cortices, posterior insula and the right DLPFC (blue color). The error bars in the graphs of the RSFC from VTA to the amygdala as well as to the motor cortex represent standard deviations. Abbr.: aHC, anterior hippocampus; Amy, amygdala; pI, posterior insula; DLPFC, dorsolateral prefrontal cortex; SMCx, sensorimotor cortex, TCx, temporal cortex.

Further widespread differences in the FC were observed from VTA to the bilateral posterior insula, sensorimotor cortex, temporal cortex, to the right DLPFC, to the left parahippocampal gyrus and bilaterally to the occipital lobe, indicating a stronger anticorrelated relationship in healthy controls than in MDD patients (Fig. 2). Similar group differences in the anticorrelated FC were detected for the substantia nigra (Table 1).

### Effect of antidepressant medication on RSFC of midbrain/brainstem nuclei

#### 5-HT nuclei

**Figure 3:**
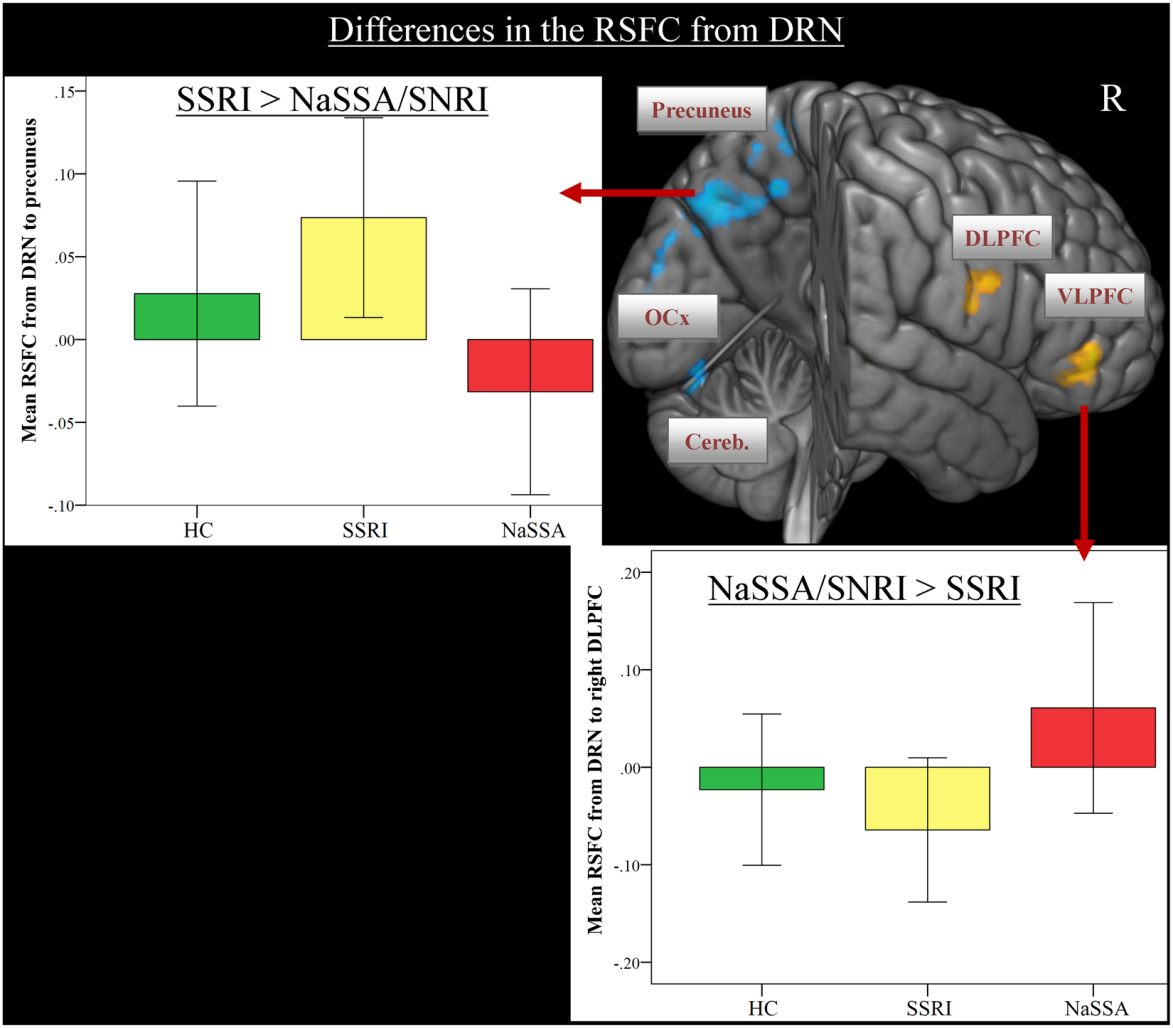
Differences between the classes of antidepressant medication in the whole-brain resting-state functional connectivity maps with seed region in the dorsal raphe nucleus (DRN). Depressed patients with an SSRI demonstrated stronger FC from DRN to the posterior brain regions in contrast to patients taking an SNRI/NaSSA (blue color). In the opposite contrast, patients with an SNRI/NaSSA showed stronger FC to the right DLPF and VLPFC (yellow color). Abbr.: SSRI, selective serotonin re-uptake inhibitor; SNRI, serotonin and noradrenalin reuptake inhibitor; NaSSA, noradrenergic and specific serotonergic antidepressant; DLPFC, dorsolateral prefrontal cortex; VLPFC, ventrolateral prefrontal cortex; OCx, occipital cortex; Cereb., cerebellum.

When comparing patients treated with an SSRI to patients treated with a NaSSA or an SNRI, significantly stronger RSFC was observed from DRN to posterior brain regions, i.e. to the precuneus, angular gyrus, occipital lobe and bilateral cerebellum (Fig. 3, Table S1). On the other hand, the NaSSA/SNRI group revealed stronger RSFC from DRN to the right DLPFC, VLPFC and bilateral superior temporal cortex (Fig. 3, Table S1).

#### Noradrenergic LC

**Figure 4:**
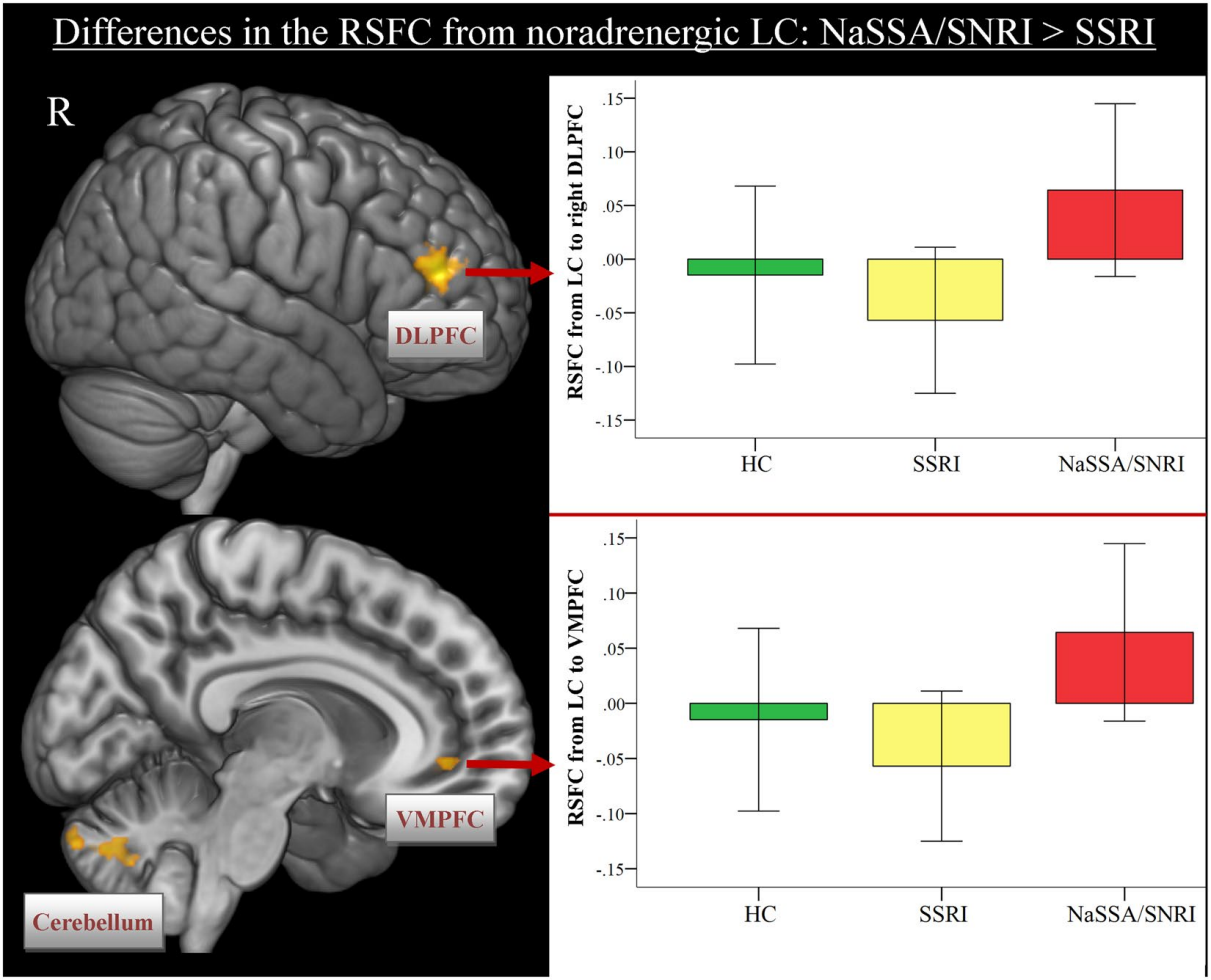
Differences between the classes of antidepressant medication in the whole-brain resting-state functional connectivity maps with seed region in the locus coeruleus (LC). Depressed patients with an SNRI or NaSSA antidepressant demonstrated stronger FC from LC to the DLPFC, VMPFC and cerebellum in contrast to patients taking an SSRI. Abbr.: SSRI, selective serotonin re-uptake inhibitor; SNRI, serotonin and noradrenalin reuptake inhibitor; NaSSA, noradrenergic and specific serotonergic antidepressant; DLPFC, dorsolateral prefrontal cortex; VMPFC, ventromedial prefrontal cortex.

Stronger connectivity was observed in the NaSSA/SNRI group from LC to bilateral DLPFC, VMPFC, inferior temporal gyrus and bilateral cerebellum (Fig. 4, Table S1). The SSRI group revealed stronger RSFC from LC to the occipital lobe, left precentral gyrus and to the parahippocampal gyrus (Table S1).

#### DA nuclei

A significantly stronger RSFC was detected in the NaSSA/SNRI group from the VTA to the left insula and bilateral cerebellum, whereas the SSRI group showed stronger RSFC to the precuneus, left inferior parietal lobule (IPL) as well as bilaterally to the middle/inferior temporal gyrus (Table S1).

## Discussion

To the best of our knowledge, this is the first time that the functional connectivity of the midbrain and upper brainstem nuclei has been studied in patients with MDD during a resting state condition.

The key finding is that MDD patients show reduced RSFC from the VTA to the dACC, mediodorsal thalamus and cerebellum, which was not significantly different regarding the type of antidepressant treatment (Fig. 1). On the other hand, patients show a significantly stronger RSFC from the VTA to the left amygdala and left DLPFC, as well as a marked pattern of absent “anti-correlation” to the posterior insula (pI), and to the sensorimotor cortex. With regard to the serotonergic and noradrenergic nuclei, relatively small group differences in the RSFC to the temporal cortex, cerebellum and sensorimotor cortex were detected.

As demonstrated previously^24^ as well as in the present study, the dopaminergic VTA has strong FC to core regions of the DMN. It is also integrated within the salience network, consistently identified using ICA of resting-state fMRI data^2, 10^. The major nodes of the salience network are the anterior insula (aI) and dACC, but include VTA (and substantia nigra) and distinct limbic areas such as the amygdala, ventral striatum, mediodorsal thalamus and hypothalamus^25^.

There is convincing evidence for the notion that the salience network plays a central role in detecting emotional and motivational salience, which triggers subsequent switching between large-scale brain networks involved in either externally- (ECN) or internally-focused (DMN) processes^25–27^. Dopaminergic projections from the VTA appear to play an important role in salience encoding^28^. Importantly, whereas the FC from VTA to dACC, mediodorsal thalamus and cerebellum was decreased in MDD patients in the present study, it was increased to the left amygdala and left DLPFC in patients when compared to healthy controls.

An abnormal functioning of several salience network nodes was often shown by previous resting-state studies in MDD^3, 4, 29^. In our recent study, we also observed an abnormal activation of the VTA, amygdala, ACC, pI and striatum during evaluation of affective and non-affective self-referential stimuli in patients suffering from MDD^30^. We would like to speculate that stronger VTA connectivity to the left amygdala and left DLPFC may indicate an over-attribution of meaning and affective salience to internal events, i.e. negative depressive thinking during the resting state condition, which may trigger associated emotional control processes. Studies of different kinds of manipulation of emotion regulation have shown that in particular lateral prefrontal regions, e.g. the DLPFC, influencing emotional responses by modulating brain regions like the amygdala^31^. The DLPFC is thought to play a crucial role in terms of top-down regulation of this affective circuitry. In MDD, hyperactivity of the amygdala was frequently observed during processing of negative stimuli^23, 32^. An interaction between increased amygdala activation during self-relevant processing and abnormal DLPFC-BOLD activation has also been demonstrated^23, 33^. Thus, our results further suggest that aberrant salience network activation and its aberrant functional connectivity may promote ruminative thinking and attentional biases toward negative events in MDD.

A further marked observation was an absent “anti-correlation” from VTA to large cluster spanning bilateral somatosensory regions and the posterior insula. The role of pI was associated with processing of somatosensory and interoceptive information, but also with evaluating motivational significance^34^. The pI was found to be anatomically and functionally connected to primary and secondary motor and somatosensory cortices^35^. We may speculate that the observed pattern of absent connectivity from VTA/SNc to sensorimotor regions and pI might be associated with the often observed motivation/volition impairments and deficits in behavioral activation, e.g. psychomotor retardation in depressed patients. Furthermore, neuroimaging studies emphasized a pivotal role of dACC in cognitive control functions^36^. Considerable evidence also exists for the important involvement of dACC in reward processing^37^ and in effort allocation integrating information about the costs and benefits of specific actions^38^. For MDD, there is strong evidence for the association between aberrant DA system and decreased reward-seeking behavior and exertion of effort^39^. The detected significant negative correlation between the functional connectivity strength from the VTA to dACC and only the BDI-II somatic factor supports this interpretation. The somatic factor was computed according to Vanheule, *et al*.^22^ and is composed of BDI-II items assessing depressive symptoms such as fatigue, appetite disturbance, loss of sexual interest and concentration difficulties.

To summarize, abnormal functional connectivity of the VTA (and in part of the substantia nigra) within the salience network may result on the one hand in an over-attribution of the affective salience of internally-oriented processes and on the other hand may be associated with diminished volition, behavioral activation and effort expenditure. Finally, our results also demonstrate that the FC from VTA and substantia nigra to DMN regions was not significantly different between medicated patients and healthy controls.

### Differential effects of antidepressant medication

Testing the impact of the class of antidepressant medication on the FC of main neurotransmitter producing nuclei, we predominantly observed a stronger FC from LC and DRN bilaterally to the DLPFC and VLPFC in patients taking 5-HT/NA antidepressant medication in contrast to patients taking SSRI only. We also observed stronger FC between LC and VMPFC in the SNRI/NaSSA group. Patients taking SSRIs showed stronger FC from DRN to posterior brain regions, i.e. superior parietal lobe, precuneus, occipital lobe and cerebellum.

The frontal lobe and the cingulate cortex have been shown to contain the highest density of NA fibers of all neocortical areas^40^, which enable the modulation of cognitive flexibility and executive functioning of this brain network^41^. Previous studies manipulating the NA neurotransmission demonstrated increased activation of the DLPFC during a working memory task after administration of atomoxetine^42^. Modafanil administration was associated with increased task-related LC and PFC activity, and enhanced LC-PFC functional connectivity^43^. Posner, *et al*.^44^ found in a 10-week double-blind, placebo-controlled trial of duloxetine, an SNRI a significant decrease in the FC between PCC and right parietal cortex as well as right superior frontal and right inferior temporal gyrus after duloxetine treatment in dysthymic patients. Furthermore, acute administration of noradrenalin reuptake inhibitors (NRI) increased DLPFC activation during processing of emotional pictures^45^. Thus, the observed pattern of increased FC from LC and DRN to prefrontal regions in our study might indicate stronger noradrenergic influence on these connections in the SNRI/NaSSA group. It is well known, that 5-HT raphe and LC neurons reciprocally influence each other^46^.

On the other hand, the effect of SSRIs has been shown in depressed patients and healthy controls on the amygdala and hippocampus activation during a cognitive task^47^ or during processing of emotional stimuli, e.g. fearful faces^45, 48^, suggesting a modulation of attentional processes by SSRIs^49^.

Here, we observed stronger connectivity in the SSRI group between LC and parahippocampal gyrus compared to the SNRI/NaSSA group. Further marked FC group differences indicate putative specific effect of SSRIs on a neural network, comprising mainly occipital and parietal areas, strongly involved in visual and attentional processes^50^. Cullen, *et al*.^51^ investigated RSFC of the amygdala in adolescents with MDD before and after 8 weeks of antidepressant treatment with an SSRI. The authors found that treatment response after 8 weeks was associated with decreased amygdala RSFC with the right precuneus and right PCC. In the same vein Wang, *et al*.^52^ reported increased amplitude of low-frequency fluctuations (ALFF) in the occipital cortex of MDD patients, who were SSRI responders in contrast to non-responders. In a very recent study Cheng, *et al*.^53^ demonstrated a decrease in the fractional ALFF in the occipital cortex 5 h after escitalopram admistration, which was a predictor of clinical remission after 8 weeks of treatment. The authors also detected increases in fractional ALFF in DLPFC, dorsomedial PFC and ACC after escitalopram admistration. Recently, Sikora, *et al*.^54^ found in a placebo-randomized controlled trial with 10 weeks open-label antidepressant treatment (mainly with citalopram) that increased baseline RSFC of the rostral ACC with the salience network (including the midbrain) was positively correlated with the response to ten weeks of antidepressant treatment. The effect of successful treatment with SSRIs on the activity of the salience network was also shown in a recent meta-analysis including positron emission tomography (PET) and anterior spin labeling fMRI (ASL-fMRI) studies^55^. It will be therefore interesting to investigate the relationship between antidepressant treatment response and changes in the functional connectivity between VTA/SNc and the salience network in a longitudinal study.

Thus, the detected differences in the FC of the midbrain/brainstem nuclei between two classes of antidepressants suggest differential neural effects of SSRIs and SNRIs. However, our interpretation is limited due to the lack of longitudinal data and data on treatment responses. To sum up, present results might suggest a possible role of RSFC of the midbrain/brainstem nuclei as a diagnostic neurobiomarker to evaluate the effects of antidepressant medication on specific neural circuitries.

Some limitations should be acknowledged. This investigation was performed as a naturalistic, non-randomized study potentially associated with a selection bias. However, both medication groups did not significantly differ regarding the severity of depression as assessed by HRSD and BDI, illness duration as well as regarding age and gender. Thus, a potential selection bias is rather unlikely. Furthermore, we used a cross-sectional study design. Longitudinal studies are necessary to investigate dynamics of the detected altered neural networks in the whole group as well as RSFC differences in the antidepressant groups after successful therapy. Further studies should include treatment-naïve patients, to compare RSFC changes of midbrain/brainstem nuclei with the treatment response caused by antidepressants at a second point in time. An additional identification of possible biomarkers for treatment response can be realized by such longitudinal studies. Including various substances in the SNRI/NaSSA group might potentially influence results, since patients may show a poor response to one drug class and a good response to another. Future studies might also benefit from a head to head comparison of two particular drugs. However, treatment related alterations in RSFC in MDD are still not sufficiently understood due to a few studies with relatively inconsistent findings. Furthermore, the investigation of anhedonia mechanisms may be a promising area for biomarker research in MDD. Since we did not use specific anhedonia questionnaires or a reward task, our interpretation regarding the association with the abnormal VTA/SNc connectivity is rather speculative. Finally, the midbrain\brainstem nuclei are relatively small, which makes it difficult to precisely assign the detected abnormal RSFC to specific nuclei. A high-resolution fMRI might be useful to improve their functional dissociation.

## Materials and Methods

### Subjects

45 patients (33 females) who met the DSM IV criteria for MDD according to the Structured Clinical Interview (SCID) for DSM-IV Axis I disorders were recruited from the inpatient service of our department. On average, patients were 36.7 ± 12.5 years old and had a mean level of education of 10.9 ± 1.3 years. Patients’ score on the Beck Depression Inventory-Second Edition (BDI-II) was 29.7 ± 8.5 and 21.1 ± 10.2 on the Hamilton Rating Scale for Depression (HRSD). Patients with a current comorbid Axis I disorder, with a history of manic episodes or with any neurological disorder were excluded from the study. Twenty three patients (8 males and 15 females) were treated with a Noradrenergic and Specific Serotonergic Antidepressant (NaSSA) or with a Selective Serotonine Noradrenaline Reuptake Inhibitor (SNRI), i.e. mirtazapine, venlafaxine and duloxetine. Eighteen patients (4 males and 14 females) were treated with a Selective Serotonin Reuptake Inhibitors (SSRI), i.e. citalopram or escitalopram and four patients were antidepressant drug-naive. Both medication groups did not significantly differ regarding age (t[39] = 1.1, p = n.s.), gender (χ² test, p = 0.5), depression severity (BDI-II: t[39] = 1.0, p = n.s.; HRSD: t[39] = 1.9, p = n.s.) and illness duration (t[38] = 1.1, p = n.s.). Patients were stably medicated for at least 10 days.

45 control subjects (33 females) matched for age, gender and education were recruited through local newspaper advertisement. The mean age was 37.6 ± 11.8 years and the mean level of education was 11.3 ± 0.9 years. The subjects’ score on the BDI-II was 2.2 ± 2.6. Subjects with past or current neurological or psychiatric diseases according to M.I.N.I^56^ and/or first-degree relatives with Axis I psychiatric disorders were excluded from the study. None of the study participants were taking any psychopharmacological medications.

A multiple choice vocabulary test (MWT-B)^57^, a measure for premorbid intelligence, confirmed that none of the study participants was mentally retarded (patients: M_IQ_ = 108.8, SD = 13.1; controls: M_IQ_ = 111.7, SD = 12.2).

All participants were right-handed, according to the modified version of Annetts handedness inventory^58^ and provided written informed consent prior to participating in the study. The study protocol was approved by the Ethics Committee of the University Hospital of Jena and informed consent was obtained from all participants. The study was conducted according to the ethical guidelines of the current official version (from 2013) of the Declaration of Helsinki. All subjects were paid 10 Euro per hour for their participation.

### MRI Procedure

The data were collected on a 3T whole body system equipped with a 20-channel head matrix coil (MAGNETOM TIM Trio, Siemens). The whole measurement consisted of a resting state scan followed by a structural MR scan. Subjects were asked to keep their eyes closed during the whole measurement. T_2_^*^-weighted images were obtained using a gradient-echo EPI sequence accelerated by parallel imaging using GRAPPA (TR = 2520 ms, TE = 30 ms, flip angle = 90°, inter-slice gap = 0.625 mm, GRAPPA factor = 2) with 45 contiguous transverse slices of 2.5 mm thickness covering the entire brain and the lower brainstem. Matrix size was 88 × 84 pixels with in-plane resolution of 2.5 × 2.5 mm^2^ corresponding to a field of view of 220 × 210 mm. A series of 240 whole-brain volume sets were acquired in one session.

High-resolution anatomical T1-weighted volume scans (MP-RAGE) were obtained in sagittal orientation (TR = 2300 ms, TE = 3.03 ms, TI = 900 ms, flip angle = 9°, FOV = 256 mm, matrix = 256 mm × 256 mm, number of sagittal slices = 192, acceleration factor (PAT = 2) with an isotropic resolution of 1 × 1 × 1 mm^3^.

### rs-fMRI preprocessing

As applied in our previous study^10^, the normalization procedure of the midbrain/brainstem was improved using the spatially unbiased infra-tentorial template (SUIT, version 3.1)^18^ to more precisely define regions of interest for the subsequent time-series extraction. Using the SUIT toolbox, we undertook the following preprocessing steps: (i) segmentation of the whole-brain image as implemented in SPM12, (ii) cropping of the image, retaining only the cerebellum and brainstem, (iii) normalization using the DARTEL (diffeomorphic anatomical registration through exponentiated lie algebra) engine^59^ that uses gray and white matter segmentation maps produced during cerebellar isolation to generate a flowfield using Large Deformation Diffeomorphic Metric Mapping LDDMM^60^, and (iv) reslicing to a voxel size of 2 × 2 × 2 mm³. Due to the small size of brainstem/midbrain nuclei and their close anatomical location, we did not smooth the normalized images. Using AFNI (http://afni.nimh.nih.gov/afni/), linear and quadratic trends were removed. The data were filtered with a frequency-based band-pass filter (AFNI 3dBandpass), retaining frequencies in the 0.01–0.08 Hz band. Head-motion was managed using multiple regression of the 6 volume-by-volume head motion parameters derived at preprocessing.

The preprocessing of the whole brain (including brainstem and cerebellum) was performed using the SPM12 (http://www.fil.ion.ucl.ac.uk/spm) and AFNI (http://afni.nimh.nih.gov/afni/) software packages. The first five images were discarded to obtain steady-state tissue magnetization. Preprocessing included 3D motion correction, i.e. rigid body realignment to the mean of all images. Subsequently, a slice timing correction was performed to ensure that the data on each slice corresponded to the same point in time. Afterwards, a within-subject registration was performed between functional and anatomical images using SPM12. The coregistered anatomical images were segmented and functional images were then spatially normalised to the MNI space using the deformation field created during the segmentation process. The whole-brain data were smoothed using a Gaussian filter of 6 mm FWHM. Preprocessing using AFNI consisted of further additional steps: (i) removal of lineal and quadratic trends, (ii) temporal band-pass filtering, retaining frequencies in the 0.01–0.08 Hz band, (iii) removal by multiple regression of several sources of variance, i.e. head-motion parameter, CSF as well as white matter signal. Due to the controversial interpretation of the functional connectivity results using global signal regression, we avoided this step in the preprocessing of the functional data^61^.

### Definition of the brainstem seed regions

As in our recent study^10^, the upper 5-HT raphe nuclei and the noradrenergic LC were identified based on their known localization in the anatomical literature^19^ and by comparison with available atlases of the human brainstem^20^. According to this literature, two seed Raphe-ROIs of 4 mm radius were defined and comprised the Nucleus raphes dorsalis (DRN, B7, MNI-coordinates, x = 2, y = −26, z = −18) and Nucleus centralis superior (B6 + B8, MNI-coordinates, x = 0, y = −32, z = −24). The LC were made up of the left (A6, 4 × 6 × 10 mm centered at MNI-coordinates, x = −5, y = −34, z = −21) and right ROI (4 × 6 × 10 mm centered at MNI-coordinates x = 7, y = −34, z = −21) lying in the floor of the forth ventricle in the rostral pons. The location of the LC-ROI corresponded to the LC mask, derived as a probabilistic map by neuromelanin-sensitive MRI in 44 healthy adults^62^.

To study the RSFC of the DA neurotransmitter system in MDD, we focused on two midbrain cell groups, the vental tegmental area (VTA, A10) and the substantia nigra, pars compacta (SNc, A9)^63^, both projecting to a broad range of cortical and subcortical brain regions^64^. To obtain the anatomically most precise ROIs, the VTA and the SNc were manually traced based on the available atlases of the human brainstem^20, 65^. Due to high concentration of neuromelanin the pars compacta had a clear contrast to the pars reticulata of the SN^66^ as well as to the neighboring regions, i.e. red nucleus and superior cerebellar peduncle relative to which SNc is dorsolaterally lying. The boundaries of the VTA were defined laterally adjacent to the substantia nigra, and medially adjacent to the interpeduncular fossa.

### Functional Connectivity Analysis

FC analyses were carried out by correlating the regional time course, which was extracted from the selected midbrain/brainstem ROIs, against all other voxels within the brain. The functional connectivity was obtained by computing Pearson correlation coefficients. After application of Fisher z-transformation to the correlation maps, using SPM12 an ANOVA was set up with one between-subjects factor GROUP (MDD patients vs. healthy controls). Our univariate analyses were primarily focused on differences in the RSFC of the upper 5-HT nuclei, both dopaminergic ROIs and noradrenergic LC. The statistical comparisons were thresholded at an uncorrected voxel-level significance of p < 0.001 and an FDR corrected cluster-level significance of p < 0.05^67^. In a further analysis we tested for the effect of antidepressant medication on the RSFC of the midbrain/brainstem nuclei. An ANOVA was set up with a between-subjects factor GROUP having three factor levels, corresponding to MDD patients treated with SSRI (n = 18), MDD patients treated with SNRI/NaSSA (n = 23) and healthy controls (n = 45). Due the smaller sample size the statistical comparisons between both medication groups were thresholded at an uncorrected voxel-level significance of p < 0.005 and an FDR corrected cluster-level significance of p < 0.05^68^.

## Electronic supplementary material

Supplementary Information
