# Supplementary material for: Treatment Associated Changes of Functional Connectivity of Midbrain/Brainstem Nuclei in Major Depressive Disorder
Source: Sci Rep. 2017 Aug 17;7:8675. doi: 10.1038/s41598-017-09077-5 (PMC5561091; doi:10.1038/s41598-017-09077-5)
Supplement: Supplementary file 1 — Supplementary Information [file 41598_2017_9077_MOESM1_ESM.pdf]

Treatment Associated Changes of Functional Connectivity of Midbrain/Brainstem Nuclei in Major Depressive Disorder

Gerd Wagner, Feliberto de la Cruz, Stefanie Köhler, Karl-Jürgen Bär

Supplementary table S1: Comparison of the resting-state functional connectivity between patients treated with a selective serotonin re-uptake inhibitor (SSRI) and with patients treated with a serotonin and noradrenalin reuptake inhibitor (SNRI) or with noradrenergic and specific serotonergic antidepressant (NaSSA) with seed regions in the serotonergic raphe nuclei, noradrenergic locus coeruleus and dopaminergic ventral tegmental area as well as substantia nigra pars compacta.

**RSFC of the DRN: NaSSA/SNRI > SSRI p<0.005 uncorr., cluster level: p<0.05 FDR corr.**							
**Region of activation**	**Right/ Left**	**Brodmann's Area**	**Cluster size**	**MNI coordinate**			**T value**
				**x**	**y**	**z**	
Superior temporal gyrus	R	22	107	44	-26	-4	4.5
Middle frontal gyrus	R	47	150	56	42	-4	4.1
Inferior frontal gyrus	R	9	142	62	6	26	3.8
Superior temporal gyrus	L	22	109	-58	4	0	3.7
**RSFC of the DRN: SSRI > NaSSA/SNRI, p<0.005 uncorr., cluster level: p<0.05 FDR corr.**							
**Region of activation**	**Right/ Left**	**Brodmann's Area**	**Cluster size**	**MNI coordinate**			**T value**
				**x**	**y**	**z**	
Precuneus	R	7	299	0	-74	46	4.4
Superior parietal lobule	L	7	128	-2	-50	68	4.0
Angular gyrus	R	39	68	34	-56	22	3.8
Occipital lobe	R	19	95	0	-88	34	3.6
Cerebellum	R		85	42	-46	-48	4.0
Cerebellum	L		75	-10	-78	-20	3.5
**RSFC of the LC: NaSSA/SNRI > SSRI p<0.005 uncorr., cluster level: p<0.05 FDR corr.**							

Region of activation	Right/ Left	Brodmann's Area	Cluster size	MNI coordinate			T value
				x	y	z	
Middle frontal gyrus	R	46	245	46	46	14	4.8
Middle frontal gyrus	L	10	80	-34	42	-12	3.9
Inferior temporal gyrus	L	20	118	-54	-54	-16	4.4
Medial frontal gyrus	R	10	80	16	36	-10	4.3
Cerebellum	R		101	10	-72	-34	4.0
Cerebellum	R		103	26	-80	-48	4.8
**RSFC of the LC: SSRI > NaSSA/SNRI, p<0.005 uncorr., cluster level: p<0.05 FDR corr.**							
**Region of activation**	**Right/ Left**	**Brodmann's Area**	**Cluster size**	**MNI coordinate**			**T value**
				x	y	z	
Precentral gyurs	L	4	105	-26	-26	60	4.2
Precentral gyurs	L	6	102	-58	6	40	4.1
Occipital lobe	R	19	234	6	-84	32	4.0
Parahippocampal gyrus	R	30	88	24	-48	2	3.2
**RSFC of the VTA: NaSSA/SNRI > SSRI p<0.005 uncorr., cluster level: p<0.05 FDR corr.**							
**Region of activation**	**Right/ Left**	**Brodmann's Area**	**Cluster size**	**MNI coordinate**			**T value**
				x	y	z	
Cerebellum	L		248	-6	-68	-32	4.4
Cerebellum	R		129	16	-62	-24	4.1
Insula	L	13	108	-40	-2	8	4.0
**RSFC of the VTA: SSRI > NaSSA/SNRI, p<0.005 uncorr., cluster level: p<0.05 FDR corr.**							
**Region of activation**	**Right/ Left**	**Brodmann's Area**	**Cluster size**	**MNI coordinate**			**T value**
				x	y	z	
Middle temporal gyrus	L	21	102	-64	-22	-6	4.5
Inferior temporal gyrus	R	21	88	68	-12	-18	4.2

Inferior parietal lobule	L	40	213	-38	-64	40	3.9
Precuneus	R	7	97	2	-64	42	3.8
